# Supplementary material for: A deep learning approach to identify gene targets of a therapeutic for human splicing disorders
Source: Nat Commun. 2021 Jun 7;12:3332. doi: 10.1038/s41467-021-23663-2 (PMC8185002; doi:10.1038/s41467-021-23663-2)
Supplement: Supplementary file 1 — Supplementary Information [file 41467_2021_23663_MOESM1_ESM.pdf]

Supplementary Information

Deep learning approach to identify targets of a novel therapeutic for human splicing disorders.

Dadi Gao[1,2,3,8], Elisabetta Morini[1,2,8], Monica Salani[2,8], Aram J. Krauson[2], Anil Chekuri[1,2], Neeraj Sharma[4], Ashok Ragavendran[1,3], Serkan Erdin[1,3], Emily M. Logan[2], Wencheng Li[5], Amal Dakka[5], Jana Narasimhan[5], Xin Zhao[5], Nikolai Naryshkin[5], Christopher R. Trotta[5], Kerstin A. Effenberger[5], Matthew G. Woll[5], Vijayalakshmi Gabbeta[5], Gary Karp[5], Yong Yu[5], Graham Johnson[6], William D. Paquette[7], Garry R. Cutting[4], Michael E. Talkowski[1,2,3]*, Susan A. Slaugenhaupt[1,2]*

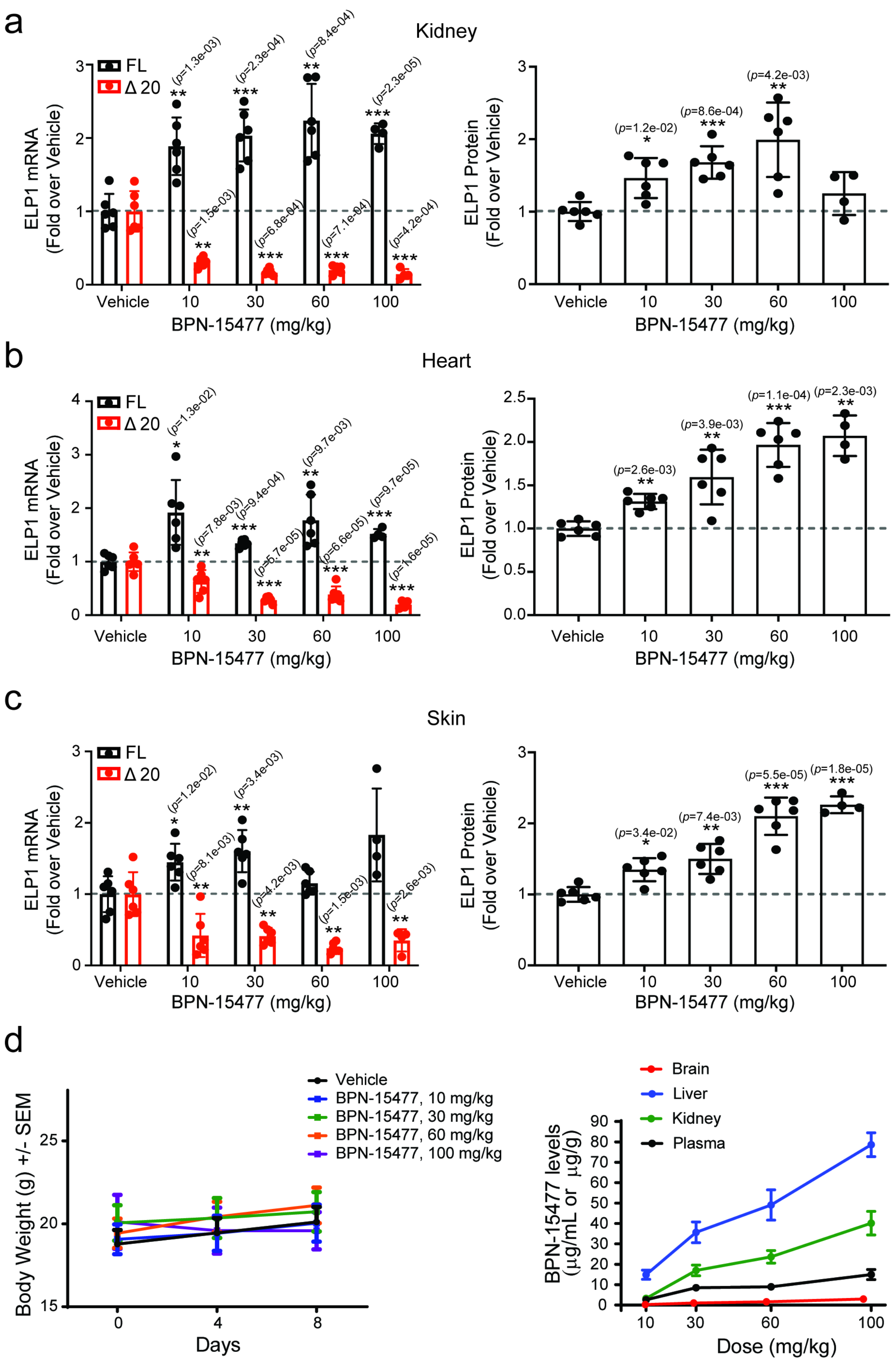
a
Kidney
ELP1 mRNA (Fold over Vehicle)
FL
Δ 20
(p=1.3e-03)
(p=2.3e-04)
(p=8.4e-04)
(p=2.3e-05)
(p=1.5e-03)
(p=6.8e-04)
(p=7.1e-04)
(p=4.2e-04)
Vehicle
10
30
60
100
BPN-15477 (mg/kg)
ELP1 Protein (Fold over Vehicle)
(p=1.2e-02)
(p=8.6e-04)
(p=4.2e-03)
b
Heart
(p=1.3e-02)
(p=7.8e-03)
(p=9.4e-04)
(p=5.7e-05)
(p=9.7e-03)
(p=6.6e-05)
(p=9.7e-05)
(p=1.6e-05)
(p=2.6e-03)
(p=3.9e-03)
(p=1.1e-04)
(p=2.3e-03)
c
Skin
(p=1.2e-02)
(p=8.1e-03)
(p=3.4e-03)
(p=4.2e-03)
(p=1.5e-03)
(p=2.6e-03)
(p=3.4e-02)
(p=7.4e-03)
(p=5.5e-05)
(p=1.8e-05)
d
Body Weight (g) +/- SEM
Days
Vehicle
BPN-15477, 10 mg/kg
BPN-15477, 30 mg/kg
BPN-15477, 60 mg/kg
BPN-15477, 100 mg/kg
Brain
Liver
Kidney
Plasma
BPN-15477 levels (μg/mL or μg/g)
Dose (mg/kg)

Supplementary Fig. 1 Bpn-15477 treatment increases full length *ELP1* transcript and protein amount in several tissues of the *TgFD9* mouse. (**a-c**) Relative expression of full-length (FL) and Δ20 *ELP1* mRNA (left graphs), and ELP1 protein quantification (right graphs) in kidney (**a**), heart (**b**) and skin (**c**) after oral doses of BPN-15477 ranging from 10 to 100 mg/kg in adult transgenic *TgFD9* mouse (n=4-6 mice in each treatment group). Comparisons are done within the same color-coded group, against the vehicle-treated mice under two-tailed Welch's *t*-test. Data are presented as mean values +/- SD. The unadjusted *p* values are displayed. (**d**) Weight assessment of *TgFD9* mice in different treatment groups. (**e**) BPN-15477 distribution in in brain, liver, kidney and plasma. The levels of compound were measured using mass spectrometry. In the figure, *$p < 0.05$; **$p < 0.01$; ***$p < 0.001$.

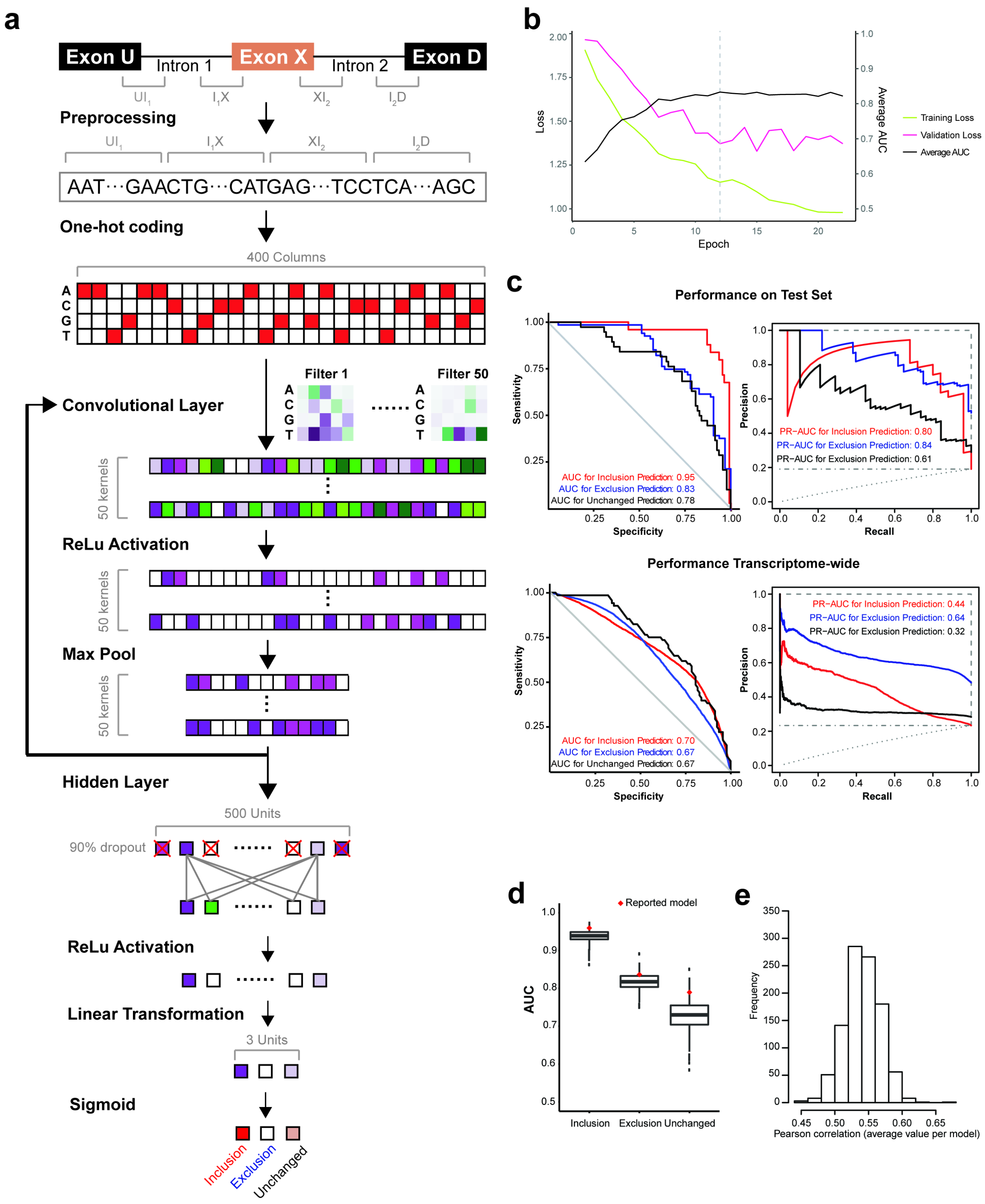

Supplementary Fig. 2 Training process of CNN

(a) CNN model workflow. For each exon-triplet, the sequences from UI_1, I_1X, XI_2 and I_2D are concatenated and then one-hot coded. In the model, two rounds of convolution were applied before

the hidden layer. Each round of convolution consists of a convolution layer of fifty filters, a ReLU activation layer and a max pooling layer of size 2. After two rounds of convolution, the output is converted and connected to a hidden layer with 90% dropout rate. The output from the hidden layer is ReLU transformed again and is then linearly transformed into a vector of three, representing three different treatment responses. The final nonlinear sigmoid maps each element in the vector to a value between 0 and 1, and it is considered as the probability of drug responsiveness. **(b)** Training progress of the CNN model. The x axis represents the number of epochs iterated during training. The left y axis shows loss score measured by binary entropy while the right y axis shows the average AUC of prediction from three classes. The red line and blue line show training and validation loss respectively along the growth of epochs. The black line shows the improvement of AUC along the growth of epochs. The vertical dashed line indicates the stop of training to avoid overfitting. **(c)** *Left panels:* ROC-AUC curves of prediction on each class using the test set and all transcriptome-wide events (see Methods), respectively. The x axis represents specificity while the y axis represents sensitivity. The solid grey line indicates the boundary beneath which the prediction is not better than a random guess. *Right panels*: PR-AUC curves prediction on each class using the test set and all transcriptome-wide events (see Methods), respectively. The x axis represents recall while the y axis represents precision. The three dashed grey lines, from top to bottom, indicate the best performance, the performance from a random classifier and the worst performance, respectively. In both panels, the performance curve for inclusion, exclusion and unchanged response are marked in red, blue and black, respectively. **(d)** AUCs for the different treatment responses obtained from 1,000 random-initiated CNN models. In the boxplot, the middle lines inside boxes indicate the medians. The lower and upper hinges correspond to the first and third quartiles. Each box extends to 1.5 times inter-quartile range (IQR)

from upper and lower hinges respectively. For each box, n=1000. Outliers were not shown. The red diamond indicates the performance of our original CNN model. **(e)** The distribution of Pearson correlation between the top thirteen most important motifs in the reported CNN model and the top thirteen most important motifs in each of the 1,000 random-initiated models.

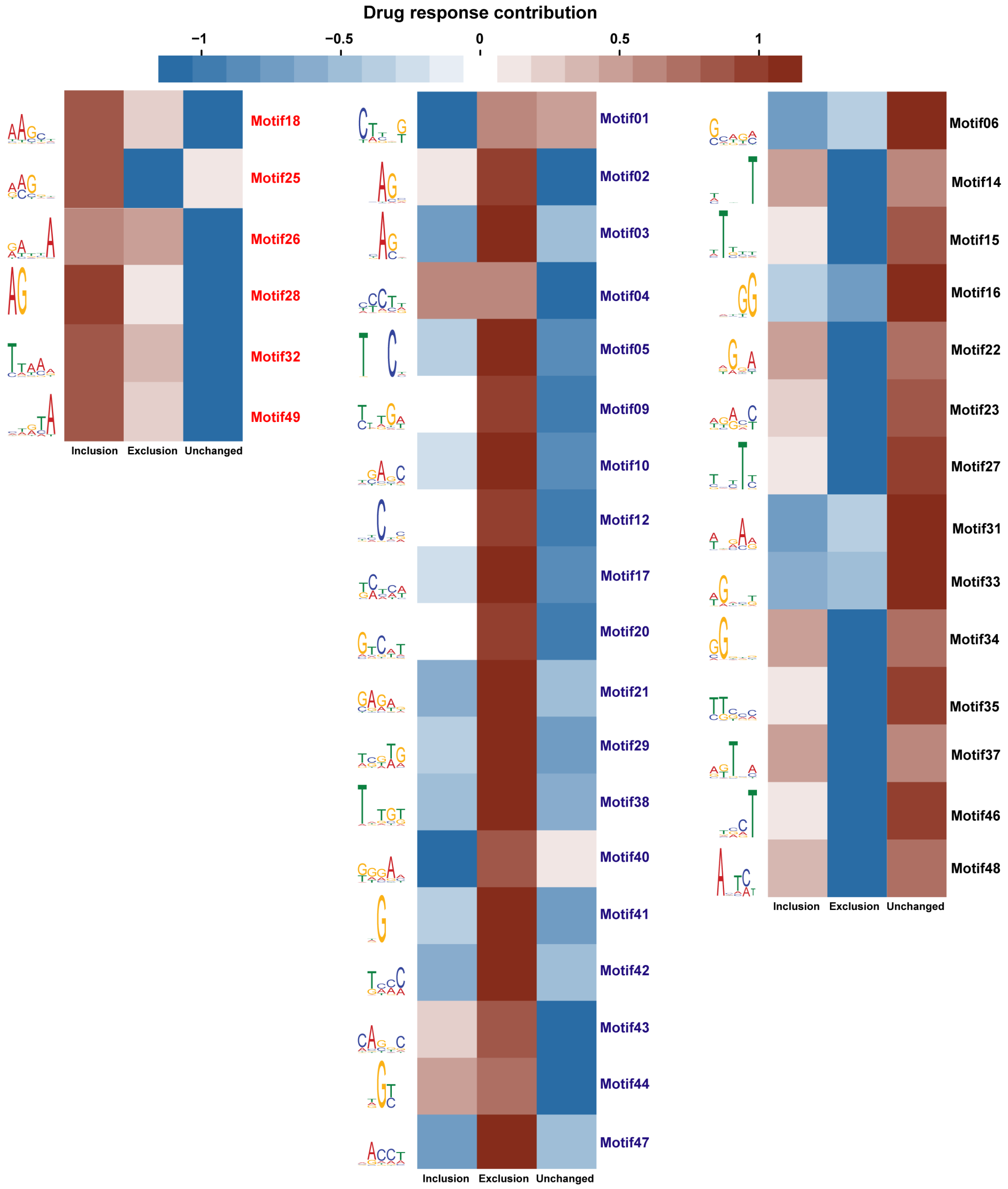

Supplementary Fig. 3 Motifs identified by the CNN model

(a) Heatmap shows all motifs identified by the model. The color indicates the directional contribution of each motif. The brown domain indicates positive contribution while the blue

domain indicates negative contribution. The LOGO plot of each motif is shown on the left side of the heatmap. The motif names are displayed on the right side of the heatmap. Colors assigned to the motif names, suggest their contribution to inclusion (red), exclusion (blue) and unchanged (black) response respectively.

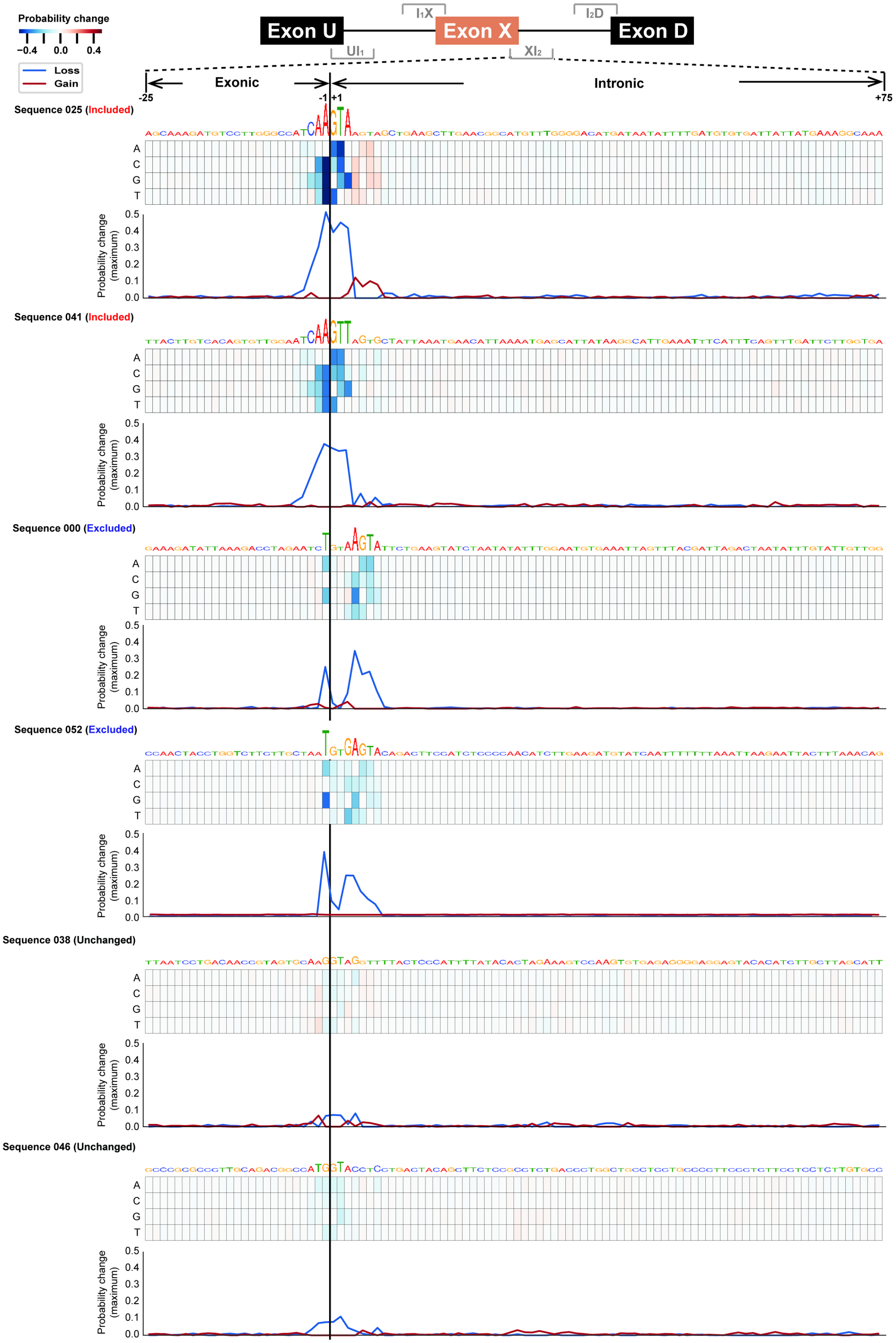

Probability change
−0.4
0.0
0.4
Loss
Gain
Exon U
Exon X
Exon D
I₁X
I₂D
UI₁
XI₂
Exonic
Intronic
-25
-1
+1
+75
Sequence 025 (Included)
Sequence 041 (Included)
Sequence 000 (Excluded)
Sequence 052 (Excluded)
Sequence 038 (Unchanged)
Sequence 046 (Unchanged)
A
C
G
T
Probability change (maximum)
0.0
0.1
0.2
0.3
0.4
0.5

Supplementary Fig. 4 *In silico* saturation mutagenesis analysis on selected sequences reveals specific patterns at the 5' splice sites of the middle exons.

The indices of the selected sequences are provided and inclusion or exclusion responses are indicated in the brackets. For each sequence, three rows are used to demonstrate the results from *in silico* saturated mutagenesis. *Upper row*: The LOGO plot represents the original input sequence before *in silico* mutagenesis while the nucleotide height represents the maximal prediction change during *in silico* mutagenesis. *Middle row*: The heatmap represents the prediction changes according to four different nucleotides. The blue color domain indicates that the new prediction has a lower score to support the class the sequence is supposed to be while the red color domain indicates the new prediction is more in favor of the class the sequence is supposed to be. The darker the color, the stronger is the prediction. The white color indicates no change in the prediction. *Bottom row*: The curve plot represents the maximal prediction change among the four nucleotides at each position.

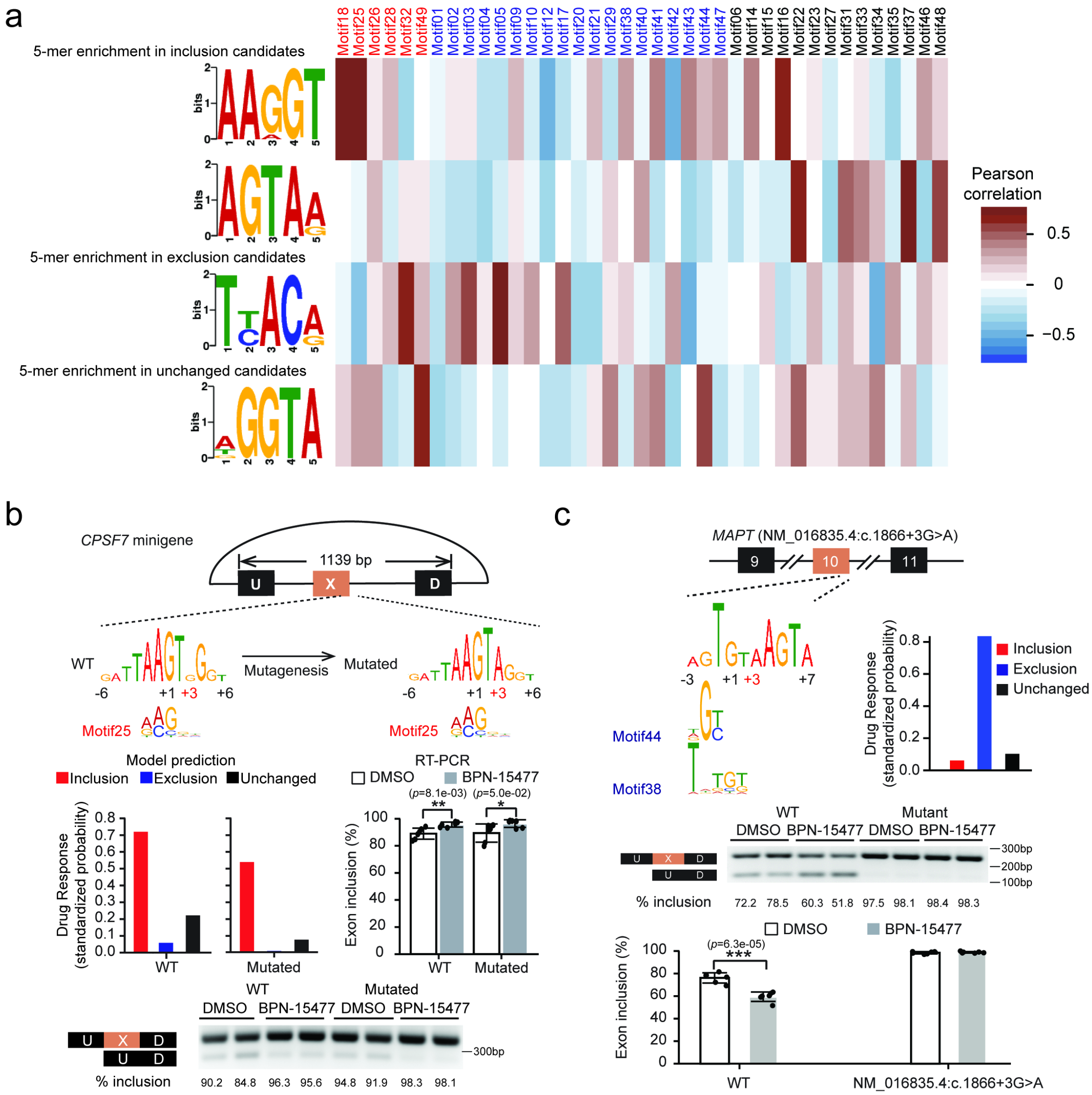

Supplementary Fig. 5 Validation of the CNN model by *k*-mer analysis, minigene with random mutagenesis, and minigene mimicking pathogenic mutation.

(a) Heatmap of Pearson correlation between the motifs identified by 5-mer enrichment analysis (each row) and the motifs identified by CNN model (each column). The brown domain indicates a positive correlation while the blue domain indicates a negative correlation. **(b)** *Upper row*: Schematic representations of *CPSF7* triplet minigene. The length of the exon triplets cloned into the minigenes are shown. The sequences adjacent to the 5' splice site of the middle exon are shown

in LOGO plots. The height of each nucleotide was estimated using *in silico* saturated mutagenesis (See Methods). The red coordinate numbers indicate the positions of mutations relative to the 5' splice sites. Their closely matched CNN motifs are indicated beneath. *Middle row*: Splicing changes of the middle exons in both wildtype and mutated exon triplets, predicted by the CNN model (*left*) and measured by RT-PCR of the minigene (*right*). To make the bar plots each experiment was repeated six times (n=6, two-tailed Welch's *t*-test). Data are presented as mean values +/- SD. The unadjusted *p* values are displayed. *Bottom row:* Example of splicing changes induced by the treatment using a minigene splicing assay. The percentage of middle exon inclusion is indicated beneath each lane. **(c)** *Upper row*: Schematic of the *MAPT* minigene construct. The sequence around the 5' splice site of the middle exon is shown in LOGO plots, with closely matched CNN motifs indicated below. The red coordinate number indicates the position of the mutation relative to the 5' splice site. The bar plots show that the CNN model predicts increased exon exclusion. *Middle row*: RT-PCR validation of treatment responses in cell lines expressing the mutated minigene. To generate the bar plots, each experiment was repeated six times. *Bottom row*: The bar plots demonstrate the splicing change promoted by BPN-15477 treatment (n=6). Data are presented as mean values +/- SD. The statistical significance is determined via two-tailed Welch's *t*-test. The unadjusted *p* values are displayed. In the figure, *$p < 0.05$; **$p < 0.01$; ***$p<0.001$.

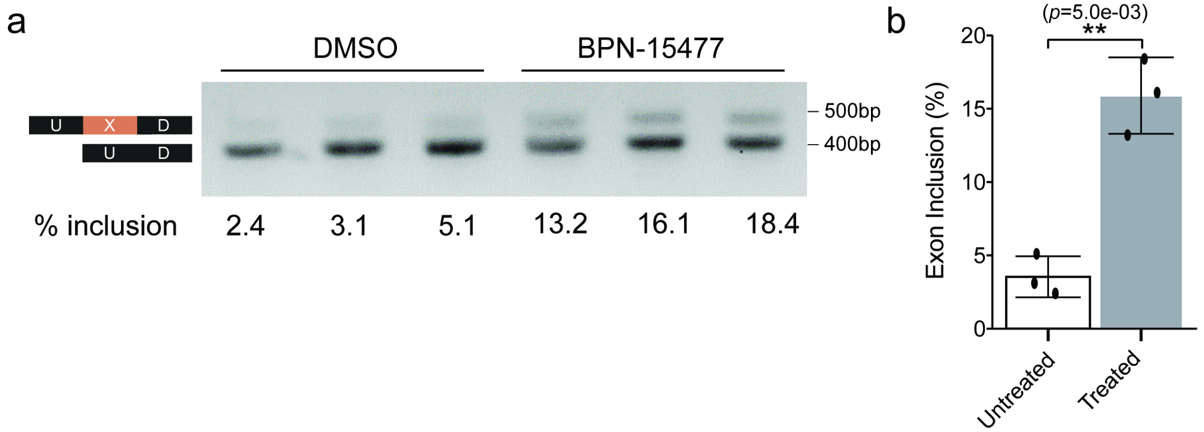

Supplementary Fig. 6 Correction of CFTR splicing in in treated CFBE cells. (a) RT-PCR validation of treatment responses in CFBE-Flpin cells stably expressing c.2988G>A-EMG-i14-i18. Cells were treated with 3 μM of BPN-15477 for three days. **(b)** The bar plots demonstrate the splicing correction promoted by BPN-15477 treatment. To generate the bar plots, the experiment was performed in triplicate (n=3). Data are presented as mean values +/- SD. The statistical significance is determined via two-tailed Welch's *t*-test. The unadjusted *p* values are displayed. In the figure, $^{**}p < 0.01$.

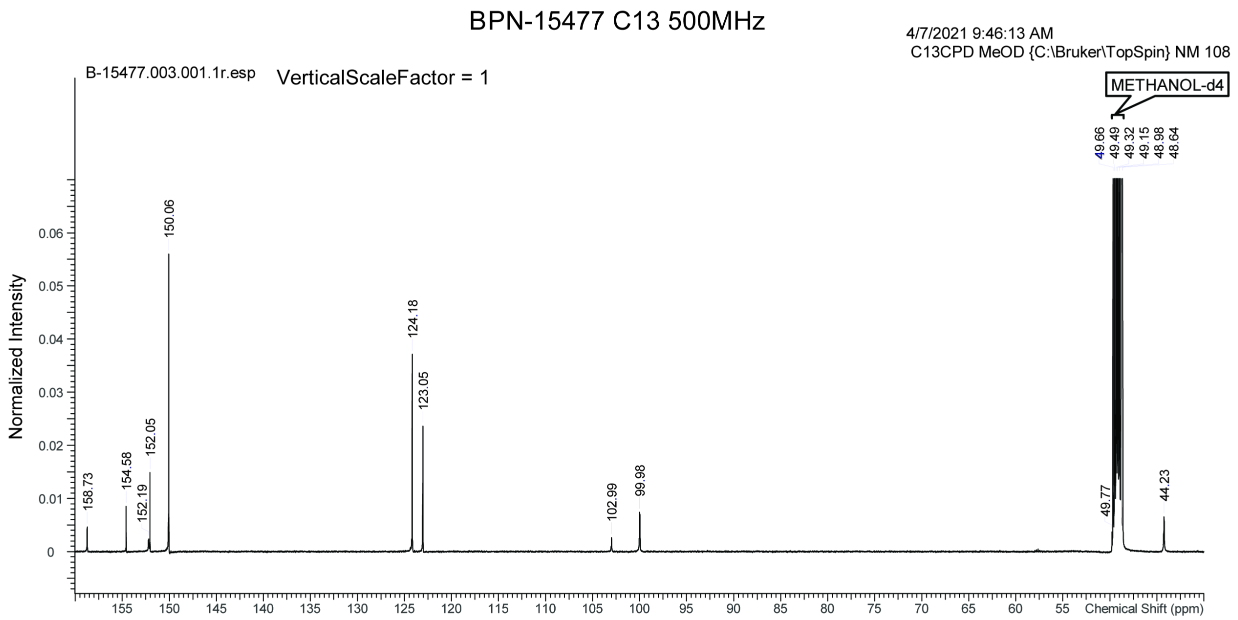

Supplementary Fig. 7 The identity and purity of the BPN-15477 established through ^{13}C-NMR.

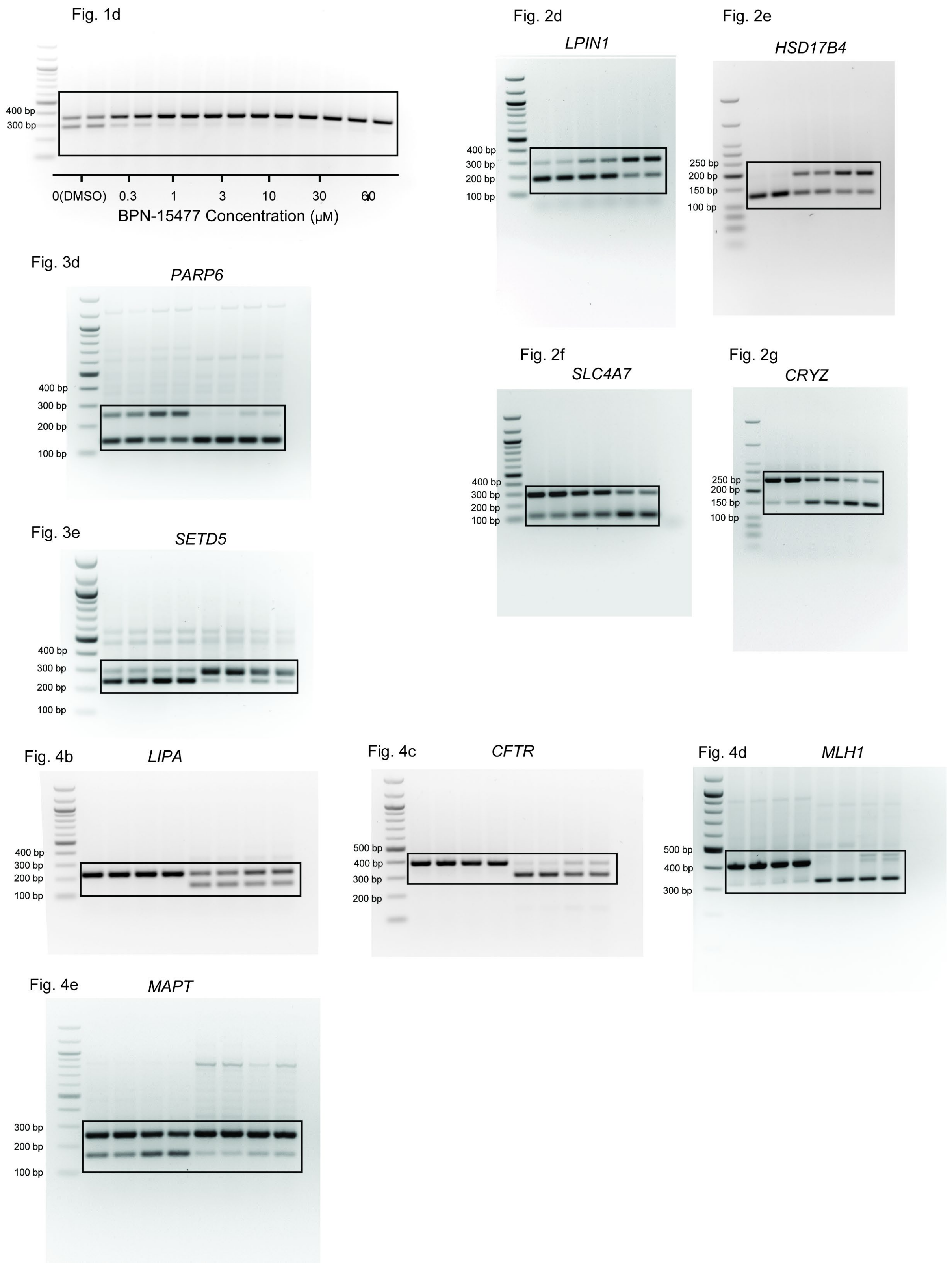

Supplementary Fig. 8 Full scan of RT-PCR gels in Figures 1-4.

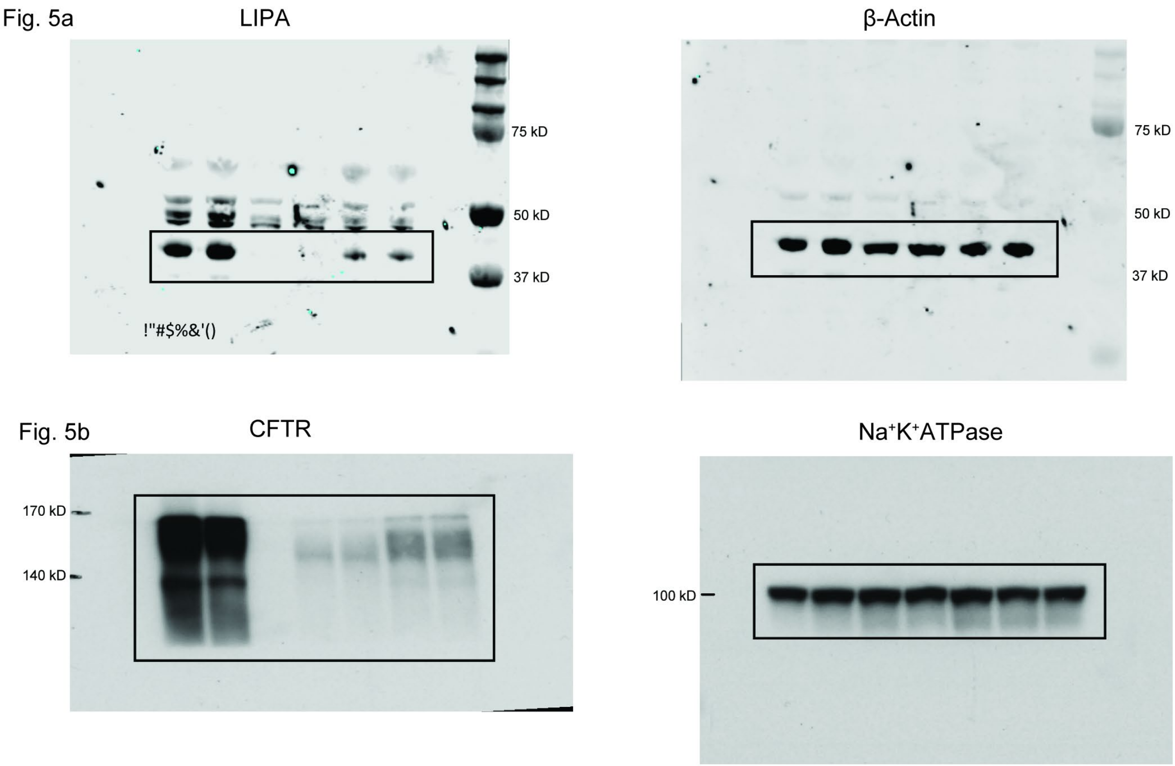

Supplementary Fig. 9 Full scan of Western blot gels in Figure 5.

Supplementary Fig. 5b

CPSF7

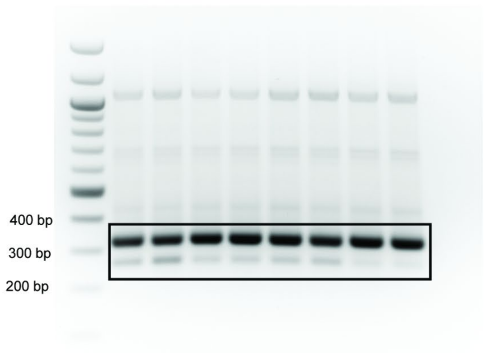

Supplementary Fig. 5c

MAPT

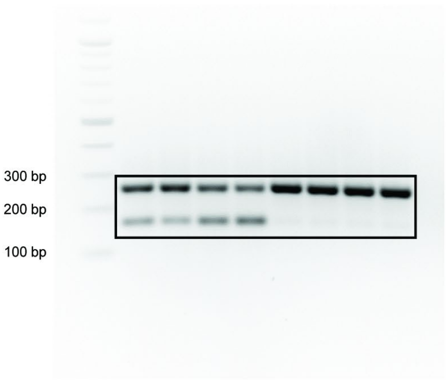

Supplementary Fig. 6

CFTR

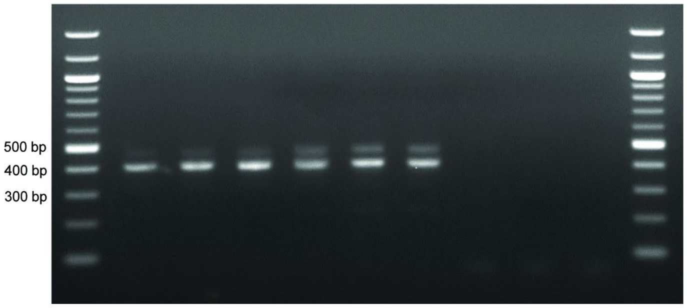

Supplementary Fig. 10 Full scan of RT-PCR gels referring to Supplementary Figures 5-6.

Supplementary Table 1 Cell lines information

Coriell #	Genotype	Sex	Age	Race
AG16409	WT	Male	12 years	Caucasian
GM03348	WT	Male	10 years	Caucasian
GM08402	WT	Male	32 years	Caucasian
GM01652	WT	Female	11 years	Caucasian
GM02036	WT	Female	11 years	Caucasian
GM00041	WT	Female	3 months	Caucasian

Supplementary Table 2 RT-PCR validation of PSI changes

Gene Symbol	PSI change in RT-PCR	PSI change in RNASeq
U2AF1L4	-0.257166667	-0.471499182
LPIN1	0.4005	0.414356642
CYLD	0.532666667	0.41517918
SLC4A7	-0.349416667	-0.426139237
CRYZ	-0.57025	-0.51621752
KLC1	0.343	0.425290445
HSD17B4	0.602666667	0.597164946
AFMID	0.453166667	0.519212881
EPB41L2	0.4412	0.410758255
CD99P1	-0.522633333	-0.457793242
LRRC28	-0.397	-0.417275893
EVC	-0.549583333	-0.496281655
MEGF6	-0.344	-0.357743325
MYEF2	0.4263	0.3531151
ASXL1	-0.0715	-0.154897036
SPTAN1	-0.1619167	-0.249074873
COPS8	0.27758333	0.230359308
PDZD11	0.3545	0.257397272
KTN1	0.11175	0.107924419
SPPL2A	0.371	0.378300362

Supplementary Table 3 Primers and melting temperature (T_m) used for RT-PCR analysis

**Gene**	**Forward 5'-3'**	**Reverse 5'-3'**	**Tm**
ELP1	CCTGAGCAGCAATCATGTG	TACATGGTCTTCGTGACATC	58 C°
KLC1	CGC AGT GGT TCC TTT AGC	CAC TGC TGC TGC TGT CG	60 C°
LPIN1	GCT GTG ATT TAC CCT CAG TCA GC	CTT AGC AGC CTG CGG CAG C	64 C°
HSD17B4	GCA GAA AGA GGA GCG TTA	GTT GGC CAC TGC TTT TCC	56 C°
SLC4A7	GCT ACA GAG GAC TGG ACG	CTA GAA CTG GAC CTG TGC TCC	60 C°
LRRC28	GAT ATA GTG CTG CAG CGT GC	CAA CTA TGT TAT TTG AGT GCA GG	60 C°
AFMID	GCC TTT CTT CCT GTT CTT TCA CG	GGT GAG CAC GTT GTC CTT CT	60 C°
CD99P1	CGA CCC AGC ACC TCT TAA TTC	CGG TGG AAT CAG GCT GCT TG	62 C°
CRYZ	GCA CTG CTG GTA CTG AGG AAG	CTT TGC CAT GGT GTC TCG TGG	64 C°
IP6K2	AAC AAG CCA AGG AGC CAA GA	ATT CAG GCC ACA CTT CCC TG	62 C°
EVC	TGC CCT GAA GCT GAT GAA GG	GGT GCC AGC GTC TGC TTC	62 C°
EPB41L2	GGA GAA GTA CCT GAT GCC GAC	CTC ACT CTC ACT GCT GCT G	60 C°
CYLD	GAT GGT TCT ACA CAG CCA CC	CTT CCC AGT AGG GTG AAG TGA C	62 C°
MEGF6	CTGGTTTGGAGAGGCCTGTG	GGGACGGACTGCAACCTCA	60 C°
MYEF2	GTACCGTGGTGCGATGACTA	TCTGACAAATATCTGGTTGCCT	60 C°
ASXL1	GCCTCGAGTTGTCCTGACTC	TTCAGGCAGGAGGAAGAGGA	60 C°
SPTAN1	CGATCGTCAGGGTTTTGTGC	TTCTGGAGCACCTCAACCTG	60 C°
COPS8	CTGAGGGACAGTCTGGGGTT	ATGGAGCAAATATAAAGCTAGAAGC	60 C°
PDZD11	GTCAGTGAGCGGAGTCTGAG	TGAGGAGGAATCCATGCTGG	60 C°
KTN1	AGGCAGAGATGGAACGATCT	TGCAAATCACCAGCTACCTTCT	60 C°
SPPL2A	TCATGGTTGAACTCGCAGCT	TGAGGCACACACTCATTACTGA	60 C°
LIPA	CCCAGAGTGCGTTTTTGAA	CCCAGTCAAAGGCTTGAAAC	60 C°
*CFTR*_HEK	GCAGTGATTATCACCAGC	GGAGGAAATATGCTCTCAAC	58 C°
PTEN	TTGCACAATATCCTTTTGAAGACCA	TTAGCATCTTGTTCTGTTTGTGGA	60 C°
CPSF7	ATTGCCCTTGACCCAGAGTT	GAGCGAATAACCTGGATCAGC	60 C°
SETD5	TGTGGTGGAATTGCCCTTAC	GCAGAATTGCCCTTCTGATA	60 C°
MLH1	GTGGAATTGCCCTTGAGC	CACTGTGCTGGATATCTGCTG	60 C°
PARP6	GGAATTGCCCTTGATCATCT	AATTGCCCTTCTGCAGTTTG	60 C°
MAPT	ACCCAAGTCGCCGTCTTCCGCC	CACCTTGCTCAGGTCAACTGGT	60 C°
*CFTR*_CFBE	GTGGCTGCTTCTTTGGTTGTG	GGAGGAAATATGCTCTCAAC	55 C°
