# Supplementary material for: A deep learning approach to identify gene targets of a therapeutic for human splicing disorders
Source: Nat Commun. 2021 Jun 7;12:3332. doi: 10.1038/s41467-021-23663-2 (PMC8185002; doi:10.1038/s41467-021-23663-2)
Supplement: Supplementary file 4 — Description of Additional Supplementary Files [file 41467_2021_23663_MOESM4_ESM.pdf]

**Title:** Supplementary Data 1

**Description:** Statistics of exon-triplets used to train the CNN model, estimated from RNASeq

**Title:** Supplementary Data 2

**Description:** Predicted targets for BPN-15477 among ClinVar pathogenic mutations
