# Supplementary material for: A deep learning approach to identify gene targets of a therapeutic for human splicing disorders
Source: Nat Commun. 2021 Jun 7;12:3332. doi: 10.1038/s41467-021-23663-2 (PMC8185002; doi:10.1038/s41467-021-23663-2)
Supplement: Supplementary file 5 — Reporting Summary [file 41467_2021_23663_MOESM5_ESM.pdf]

nature research

Corresponding author(s): Susan A. Slaugenhaupt

Last updated by author(s): Mar 30, 2021

Reporting Summary

Nature Research wishes to improve the reproducibility of the work that we publish. This form provides structure for consistency and transparency in reporting. For further information on Nature Research policies, see our Editorial Policies and the Editorial Policy Checklist.

Statistics

For all statistical analyses, confirm that the following items are present in the figure legend, table legend, main text, or Methods section.

n/a	Confirmed	
☐	☒	The exact sample size (n) for each experimental group/condition, given as a discrete number and unit of measurement
☐	☒	A statement on whether measurements were taken from distinct samples or whether the same sample was measured repeatedly
☐	☒	The statistical test(s) used AND whether they are one- or two-sided<br>*Only common tests should be described solely by name; describe more complex techniques in the Methods section.*
☐	☒	A description of all covariates tested
☐	☒	A description of any assumptions or corrections, such as tests of normality and adjustment for multiple comparisons
☐	☒	A full description of the statistical parameters including central tendency (e.g. means) or other basic estimates (e.g. regression coefficient) AND variation (e.g. standard deviation) or associated estimates of uncertainty (e.g. confidence intervals)
☐	☒	For null hypothesis testing, the test statistic (e.g. F, t, r) with confidence intervals, effect sizes, degrees of freedom and P value noted<br>*Give P values as exact values whenever suitable.*
☒	☐	For Bayesian analysis, information on the choice of priors and Markov chain Monte Carlo settings
☐	☒	For hierarchical and complex designs, identification of the appropriate level for tests and full reporting of outcomes
☐	☒	Estimates of effect sizes (e.g. Cohen's d, Pearson's r), indicating how they were calculated

Our web collection on statistics for biologists contains articles on many of the points above.

Software and code

Policy information about availability of computer code

Data collection: No software was used for data collection.

Data analysis:
GraphPad Prism 9
STAR v2.5.2a [https://github.com/alexdobin/STAR/archive/refs/tags/2.5.2a.tar.gz];
Basset [https://github.com/davek44/Basset];
MEME v4.11.2 [https://meme-suite.org/meme/meme-software/4.11.2/meme_4.11.2.tar.gz];
SpliceAI [https://github.com/illumina/SpliceAI];
Custom codes [https://github.com/talkowski-lab/SMC_CNN_Model]

For manuscripts utilizing custom algorithms or software that are central to the research but not yet described in published literature, software must be made available to editors and reviewers. We strongly encourage code deposition in a community repository (e.g. GitHub). See the Nature Research guidelines for submitting code & software for further information.

Data

Policy information about availability of data

All manuscripts must include a data availability statement. This statement should provide the following information, where applicable:
- Accession codes, unique identifiers, or web links for publicly available datasets
- A list of figures that have associated raw data
- A description of any restrictions on data availability

The datasets generated during and/or analysed during the current study are available in the GEO database:
GSE158947 [https://www.ncbi.nlm.nih.gov/geo/query/acc.cgi?acc=GSE158947]
Other public datasets used in this study:

Human genome Ensembl GRCh37 [http://ftp.ensembl.org/pub/release-75/fasta/homo_sapiens/dna/Homo_sapiens.GRCh37.75.dna.primary_assembly.fa.gz]
Human transcriptome Ensembl GRCh37.75 [http://ftp.ensembl.org/pub/release-75/gtf/homo_sapiens/Homo_sapiens.GRCh37.75.gtf.gz]
ClinVar GRCh37 VCF version 20190325 [https://ftp.ncbi.nlm.nih.gov/pub/clinvar/vcf_GRCh37/archive_2.0/2019/clinvar_20190325.vcf.gz]
gnomAD v2.11 GRCh37 VCF [https://gnomad.broadinstitute.org/downloads#v2-variants]

Field-specific reporting

Please select the one below that is the best fit for your research. If you are not sure, read the appropriate sections before making your selection.

☒ Life sciences ☐ Behavioural & social sciences ☐ Ecological, evolutionary & environmental sciences

For a reference copy of the document with all sections, see nature.com/documents/nr-reporting-summary-flat.pdf

Life sciences study design

All studies must disclose on these points even when the disclosure is negative.

Sample size	Power analysis was used to determine the sample sizes for this study.
Data exclusions	No data were excluded from the analysis.
Replication	The Dual luciferase assay was independently repeted three time. Each experimental validation using RT-PCR was repeated six times, except the one referring to Supplementary Figure 6a was repeated three times. The specific number of replicates for each experiment is detailed in the figure legend. The protein quantifications using western blot were interdependently repeated six times. CFTR chloride channel analysis in CFBE-Flpin cells was repeated at least two times. All the attempts at replication were successful.
Randomization	Samples were randomly assigned to the vehicle or treated group.
Blinding	Investigators conducting the experiments were blind to treatment category.

Reporting for specific materials, systems and methods

We require information from authors about some types of materials, experimental systems and methods used in many studies. Here, indicate whether each material, system or method listed is relevant to your study. If you are not sure if a list item applies to your research, read the appropriate section before selecting a response.

Materials & experimental systems

n/a	Involved in the study	
☒	☐	Antibodies
☐	☒	Eukaryotic cell lines
☒	☐	Palaeontology and archaeology
☐	☒	Animals and other organisms
☒	☐	Human research participants
☒	☐	Clinical data
☒	☐	Dual use research of concern

Methods

n/a	Involved in the study	
☒	☐	ChIP-seq
☒	☐	Flow cytometry
☒	☐	MRI-based neuroimaging

Eukaryotic cell lines

Policy information about cell lines

Cell line source(s)	All the cell lines used in this study were purchased either from ATCC (HEK293) or Coriell Institute (AG16409, GM03348,GM08402,GM01652,GM02036,GM00041, GM04663 and GM03111). The Flp-In 293 cells stably expressing the CFTR minigene carrying the c.2988G>A mutation (EMG-MUT) were kindly provided by Dr. Garry R. Cutting at Johns Hopkins University school of Medicine.
Authentication	The Flp-In 293 cells (R75007, ThermoFisher Scientific) stably expressing the minigene containing the CFTR c.2988G>A mutation were previously authenticated by Dr. Cutting team as described in Neeraj Sharma et al, Hum Mutat. 2014. The remaining cell lines are commercially available and they were authenticated by the cell repositories of origin using G-banded karyotyping.
Mycoplasma contamination	All cell lines were tested negative for mycoplasma.
Commonly misidentified lines (See ICLAC register)	No misidentified lines were used in this study.

Animals and other organisms

Policy information about studies involving animals; ARRIVE guidelines recommended for reporting animal research

Laboratory animals	C57BL/6 mice of both sex not older than 3 months of age.
Wild animals	NA
Field-collected samples	NA
Ethics oversight	All experimental protocols were approved by the Institutional Animal Care and Use Committee of the Rutgers University, and were in accordance with NIH guidelines.

Note that full information on the approval of the study protocol must also be provided in the manuscript.
